# Mapping the scientific research on exercise therapy for Alzheimer’s disease: a scientometric study of hotspots and emerging trends

**DOI:** 10.3389/fnagi.2025.1536515

**Published:** 2025-04-03

**Authors:** Shi Long Song, Wen Bing Yu, Xin Min Cai, Jie Ma, Lu Lu Zou, Li Li Gao, Shi Ming Li

**Affiliations:** ^1^Department of Neurology, Qingdao Hospital of Traditional Chinese Medicine (Qingdao Hiser Hospital), Qingdao, Shandong, China; ^2^Teaching Center of Fundamental Courses, Ocean University of China, Qingdao, Shandong, China; ^3^Qingdao Hong Kong East Road Primary School, Qingdao, Shandong, China

**Keywords:** Alzheimer’s disease, exercise therapy, visual analysis, research hotspots, emerging trends

## Abstract

**Background:**

Alzheimer’s disease (AD) is the most common form of dementia globally, placing a substantial economic burden on patients and society. Exercise serves as an adjuvant therapy for AD with a low incidence of related adverse events. As a non-pharmacological intervention, it has demonstrated significant potential in the therapy of AD.

**Objective:**

This study examines the key hotspots and emerging trends in exercise therapy for AD, offering valuable insights for researchers engaged in future research in this field.

**Methods:**

The Web of Science Core Collection database was utilized to search for literature on exercise therapy for AD from January 1, 2000, to November 1, 2024, with 1,372 relevant articles being identified. And CiteSpace 6.2.R4 and VOSviewer were used to evaluate the bibliometric indicators.

**Results:**

Since 2000, the number of publications in the field of exercise therapy for AD has been increasing. In addition to the well-known areas of research, such as the impact of exercise on cognitive function, the combination of exercise and medication therapy, the effects of exercise on specific symptoms, and the exercise with music therapy, the field may also focus on more novel topics. These include the integration of the design and implementation of exercise interventions, exercise and dendritic spines, and exercise and neurophysiological mechanisms. Furthermore, an analysis of emerging keywords reveals that the current main research direction is exploring the specific physiological mechanisms through which exercise affects AD. This includes three emerging trends: the impact of exercise on cognitive impairment in AD patients, the effects of exercise on brain-derived neurotrophic factor (BDNF) and Amyloid beta, and the influence of exercise on Stress and neuroinflammation.

**Conclusion:**

The research results indicate that interventions using exercise to alleviate the negative symptoms of AD, as well as combining exercise with medication for therapy, are gaining increasing attention from researchers. Meanwhile, novel topics such as exercise and music therapy, the design and implementation of exercise interventions, and neurophysiological mechanisms should also attract scholarly interest. Additionally, exploring the physiological mechanisms behind exercise therapy for AD could be a key focus for future research.

## 1 Introduction

AD is the most common type of dementia, a neurodegenerative disease that severely impairs cognitive functions such as memory, reasoning, and communication. One of its key characteristics is the excessive production and accumulation of amyloid protein, leading to plaque formation and cognitive impairment (Rosa and Fahnestock, 2014). There is a direct relationship between amyloid beta deposition and impaired BDNF regulation (Christensen et al., 2008). The global prevalence of AD is primarily influenced by population aging, while advancements in diagnostic technologies have led to the identification of more cases. Changes in lifestyle, unhealthy diets, lack of exercise, as well as environmental factors and genetic susceptibility, may also increase risk. Additionally, chronic conditions such as cardiovascular disease, diabetes, and obesity are associated with an increased risk of AD. According to the World Health Organization, more than 55 million people worldwide live with dementia, and this number is projected to rise to 78 million by 2030 and 139 million by 2050 (World Failing to Address Dementia Challenge, 2024). AD inflicts severe and multifaceted harm, both neurologically and socioeconomically. Recent research reveals that AD accelerates the loss of neurons, contributing to significant brain atrophy, especially in regions linked to memory and cognition. A 2024 study found that medicare payments for beneficiaries aged 65 and older with AD or other dementias are nearly three times higher than those without these conditions. Medicaid payments for these individuals are over 22 times greater. In 2024, total estimated payments for health care, long-term care, and hospice services for people aged 65 and older with dementia are projected to reach $360 billion, reflecting the substantial financial burden of dementia care on the healthcare system (Alzheimer’s Association Report, 2024). Therefore, the urgent need for effective therapeutic strategies has driven an increase in research focused on interventions that can slow the progression of AD or alleviate its symptoms. Among these interventions, exercise therapy has gained attention as a promising non-pharmacological approach, showing potential to improve cognitive function, reduce neuroinflammation, and enhance quality of life for AD patients.

Exercise therapy, including structured physical activities such as aerobic exercise, resistance training, and balance exercises, has been shown to offer multiple benefits for patients with AD. An increasing body of literature indicates that exercise not only improves overall physical health but also supports cognitive function, mental health, and quality of life in AD patients (Li et al., 2024; Hoffmann et al., 2013; Abd and Al-Jiffri, 2016). Studies have explored the neurobiological mechanisms underlying these benefits, including the role of exercise in enhancing neuroplasticity (Yogev-Seligmann et al., 2021), reducing neuroinflammation (Yuan et al., 2022), improving cerebral blood flow (Ivey et al., 2011), and increasing levels of BDNF (Marston et al., 2017). However, despite the accumulating evidence, a comprehensive analysis of exercise therapy for AD is still lacking. However, despite the continuous accumulation of evidence, there is still a lack of comprehensive analysis regarding exercise therapy for AD at present.

Given the diversity of research and the rapid growth in publications, a comprehensive and systematic analysis of the literature is necessary to map the intellectual landscape, identify key trends, and uncover gaps in the current knowledge. Bibliometric and visual analysis tools, such as CiteSpace and VOSviewer, provide an effective way to address these needs. These tools enable researchers to explore patterns in large bodies of scientific literature, revealing connections between authors, institutions, keywords, and research topics. In this study, CiteSpace and VOSviewer software were used to perform a visual analysis of the articles in the field of exercise therapy for AD from the Web of Science Core Collection database, aiming to identify research hotspots and emerging trends, and provide reference for future research and therapy of AD.

## 2 Materials and methods

### 2.1 Data source and search strategy

**Figure 1:**
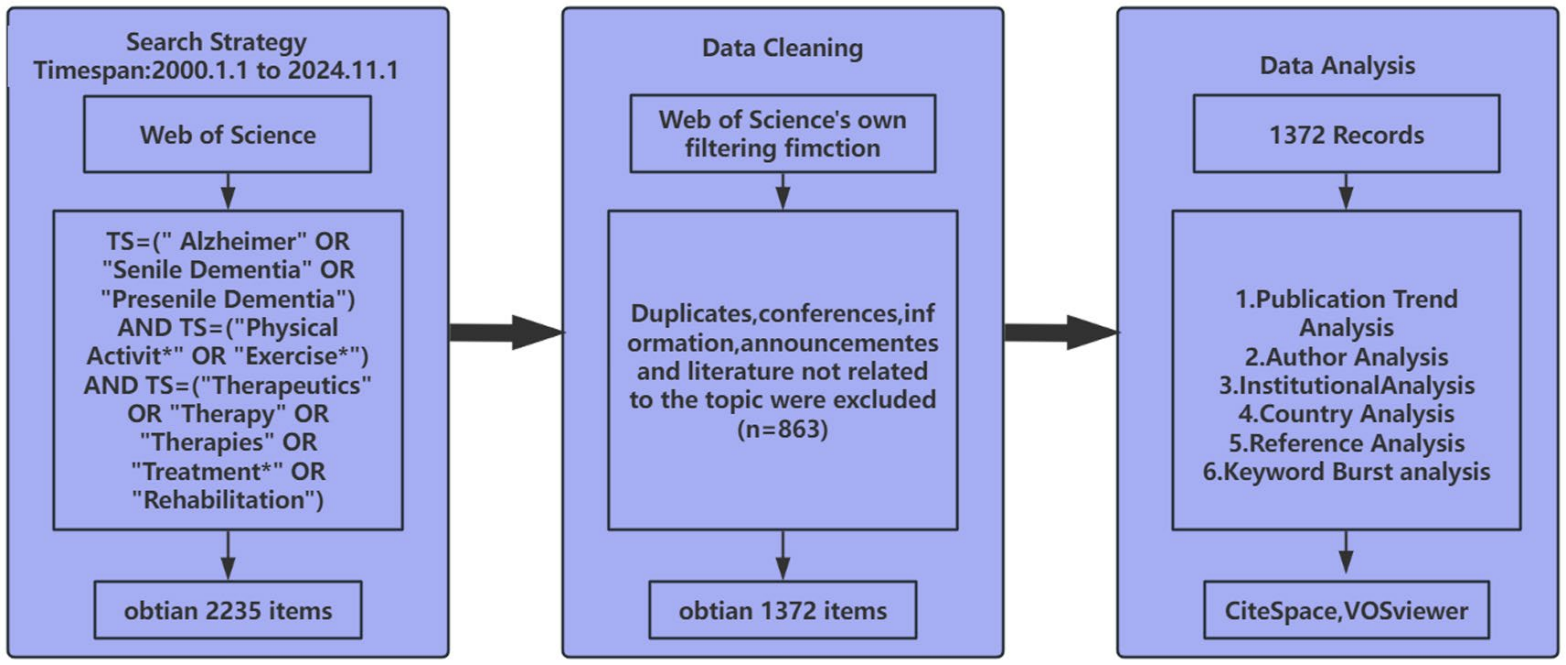
Data retrieval flow chart.

For this study, the Web of Science core collection was employed as the primary data source spanning the period from January 1, 2000, to November 1, 2024. Search for topic terms in PubMed’s Medical Subject Headings (MeSH) and confirm search terms based on expert knowledge. The adopted search strategy was formulated as: TS = (“Alzheimer” OR “Senile Dementia” OR “Presenile Dementia”) AND TS = (“Physical Activity*” OR “Exercise*”) AND TS = (“Therapeutics” OR “Therapy” OR “Therapies” OR “Treatment*” OR “Rehabilitation”). This strategy resulted in an initial yield of 2,235 documents. To guarantee the quality and reliability of the literature review, the filtering functions of the Web of Science were employed to exclude certain document types, ensuring that only articles were retained. Subsequently, a manual screening process was conducted, excluding documents that were identified as duplicates, off-topic, or failing to meet the predefined selection criteria, based on a thorough review of keywords, titles, and abstracts. Consequently, a refined data set of 1,372 articles was included in this study. For further analysis, the selected literature was saved in “full record and cited references” format as txt files for subsequent bibliometric analysis using CiteSpace and VOSviewer. The article screening process is illustrated in Figure 1. The specific exclusion criteria were: (1) articles not related to AD; (2) document types other than articles.

### 2.2 Data extraction

A standardized search strategy was employed by two researchers to extract documents, consolidating synonymous keywords. For example, variations of “exercises” and “exercise” were standardized to “exercise*” and different forms of “therapy” were unified to “therapy.” Discrepancies in keywords were resolved through discussions among the researchers and, when necessary, with the consultation of a third party. Documents were screened in batches according to the inclusion criteria to identify eligible studies. Authors were included regardless of their rank, and their contributions were referenced based on the number of publications in this study.

### 2.3 Visualization analysis method and bibliometric analysis

To conduct a bibliometric analysis of literature pertaining to exercise therapy for AD, software tools such as CiteSpace and VOSviewer were employed. These tools generated knowledge graphs, which delved into word frequency, clustering patterns, and citation analysis across various modules, including authors, countries, institutions, keywords, and references. Through this analysis, we identified prominent authors, countries, and institutions in the field since 2000. Furthermore, the dominant themes and emerging trends in the research on exercise therapy for AD were explored, providing valuable insights into potential future research directions.

## 3 Results

### 3.1 Overall characteristics of publications

**Figure 2:**
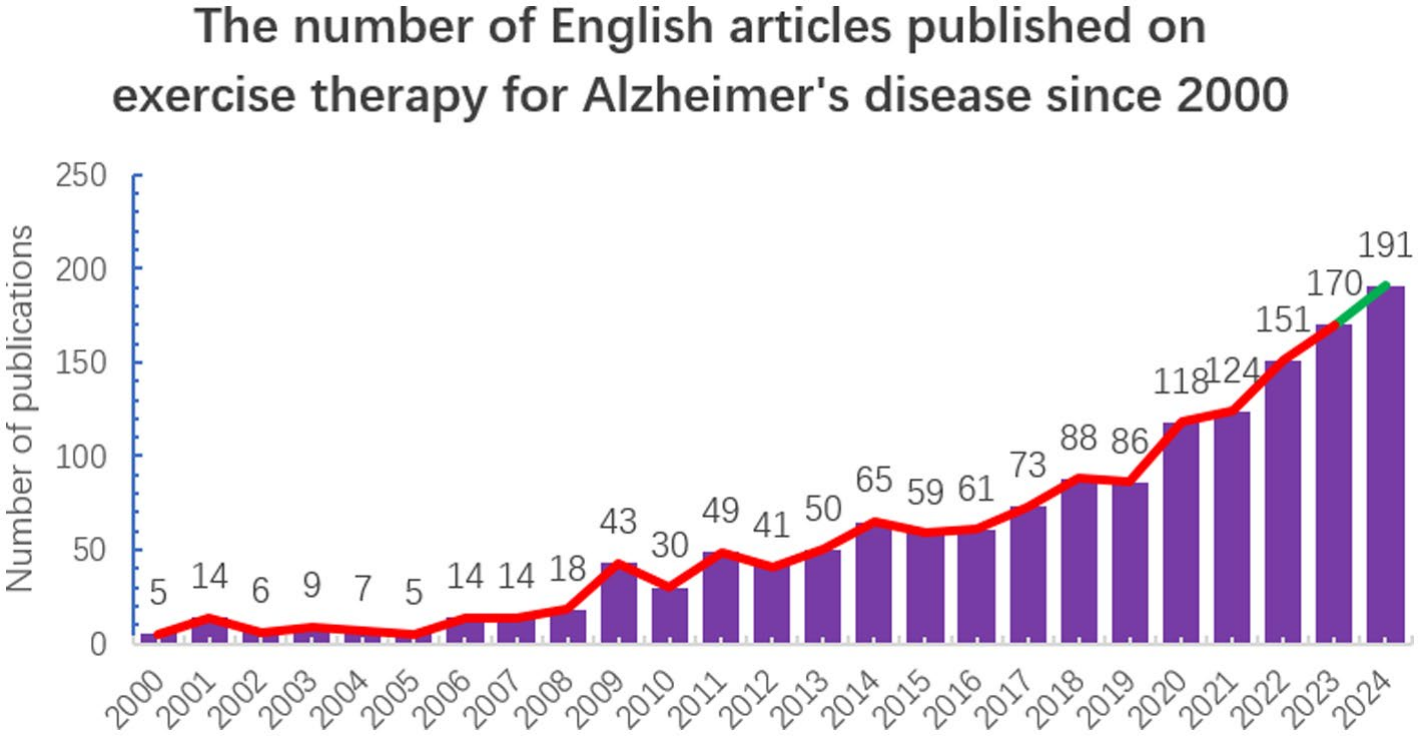
Annual number of articles published on exercise therapy for AD from 2000 to 2024.

As shown in Figure 2, research on exercise therapy for AD has shown a significant upward trend from 2000 to 2024. In the initial decade (2000–2009), this field was in its infancy, with relatively few publications and gradually increasing research output. Starting in 2010, as understanding of AD deepened, the potential benefits of exercise therapy became more apparent, particularly after Kirk I. Erickson et al. published the paper “Exercise training increases size of hippocampus and improves memory” in PNAS (Erickson et al., 2011), demonstrating that exercise can truly alter the brain. This sparked heightened research interest. Notably, in recent years (2016–2023), the field experienced substantial growth, peaking at 170 publications in 2023. This remarkable increase highlights the academic community’s growing focus on the therapeutic potential of exercise for AD, suggesting that further in-depth research and clinical development may continue in the near future. Since 2024 is not yet complete, the number of publications for 2024 is an estimate, calculated based on the average growth rate over the past five years.

### 3.2 Analysis of authors and co-cited authors

Table 1 listed the top 10 authors in the field of exercise therapy for AD research, based on their publication counts and co-citation frequencies. Hauer, Klaus and Yu, Fang lead with 10 publications each, demonstrating their significant contributions. Vellas, Bruno and Mccurry, Susan M. are the most co-cited authors for Hauer and Yu respectively, with 1,342 and 1,162 co-citations, indicating their collaborative influence. Gimenez-llort, Lydia follows closely with 9 publications and a high co-citation count of 1,108 with Andrieu, Sandrine. Other notable authors include Mccurry, Stella, Florindo, Barnes, Deborah E., Gobbi, Sebastiao, Harwood, Rowan H., Kivipelto, Miia, and Komaki, Alireza, all with 7 publications and substantial co-citation numbers, showcasing their collaborative efforts and impact on advancing the research in this vital area.

**Table 1:** The author of the research on exercise therapy for AD with the most frequent publication and co-citation.

Rank	Authors	Counts	Co-cited authors	Counts
1	Hauer, Klaus	10	Vellas, Bruno	1,342
2	Yu, Fang	10	Mccurry, Susan M.	1,162
3	Gimenez-Llort, Lydia	9	Andrieu, Sandrine	1,108
4	Mccurry, Susan M.	8	Dartigues, Jean-Francois	637
5	Stella, Florindo	8	Gimenez-llort, Lydia	541
6	Barnes, Deborah E.	7	Logsdon, Rebecca G.	495
7	Gobbi, Sebastiao	7	Teri, Linda	467
8	Harwood, Rowan H.	7	Cristofol, Rosa	447
9	Kivipelto, Miia	7	Sanfeliu, Coral	447
10	Komaki, Alireza	7	Stella, Florindo	443

### 3.3 Analysis of country

In the field of research on exercise therapy for AD, researchers from various countries have demonstrated a proactive spirit of exploration. Table 2 lists the publication volume and centrality of the top 10 countries in AD therapy research. Centrality is a network analysis metric used to assess the importance of a node within a network. Nodes with high centrality are typically considered key nodes, possessing significant influence and connectivity. This helps identify important researchers, institutions, or topics, as well as reveal research hotspots and foster collaboration opportunities. The United States (USA) stands out prominently with a remarkable 466 publications, underscoring not only the country’s profound research foundation in this domain but also its advantage in scientific research investment. China follows closely with 156 publications, and although its number falls short of that of the USA, its centrality index indicates a certain level of influence in the field. Countries such as England, Italy, Canada, and Germany have also contributed substantially to the research landscape, each with over 70 publications, demonstrating their active engagement and contributions to the study of exercise therapy for AD. Spain and Australia, on the other hand, exhibit higher centrality values, signifying that their research findings are highly recognized and influential within the field. Notably, the high network density of 0.1975 underscores the close research collaboration among countries worldwide. Overall, the research endeavors of these nations have collectively propelled the progress and development of the field of exercise therapy for AD.

**Table 2:** Top 10 countries on the research of research on exercise therapy for AD.

Rank	Country	Counts	Centrality	Number of Researchers
1	USA	466	0.38	990
2	Peoples R China	156	0.13	144
3	England	108	0.09	89
4	Italy	94	0.06	150
5	Canada	89	0.06	149
6	Germany	79	0.09	126
7	Spain	78	0.21	135
8	Australia	77	0.1	138
9	Japan	71	0.02	115
10	South Korea	68	0.06	106

### 3.4 Analysis of institution

**Figure 3:**
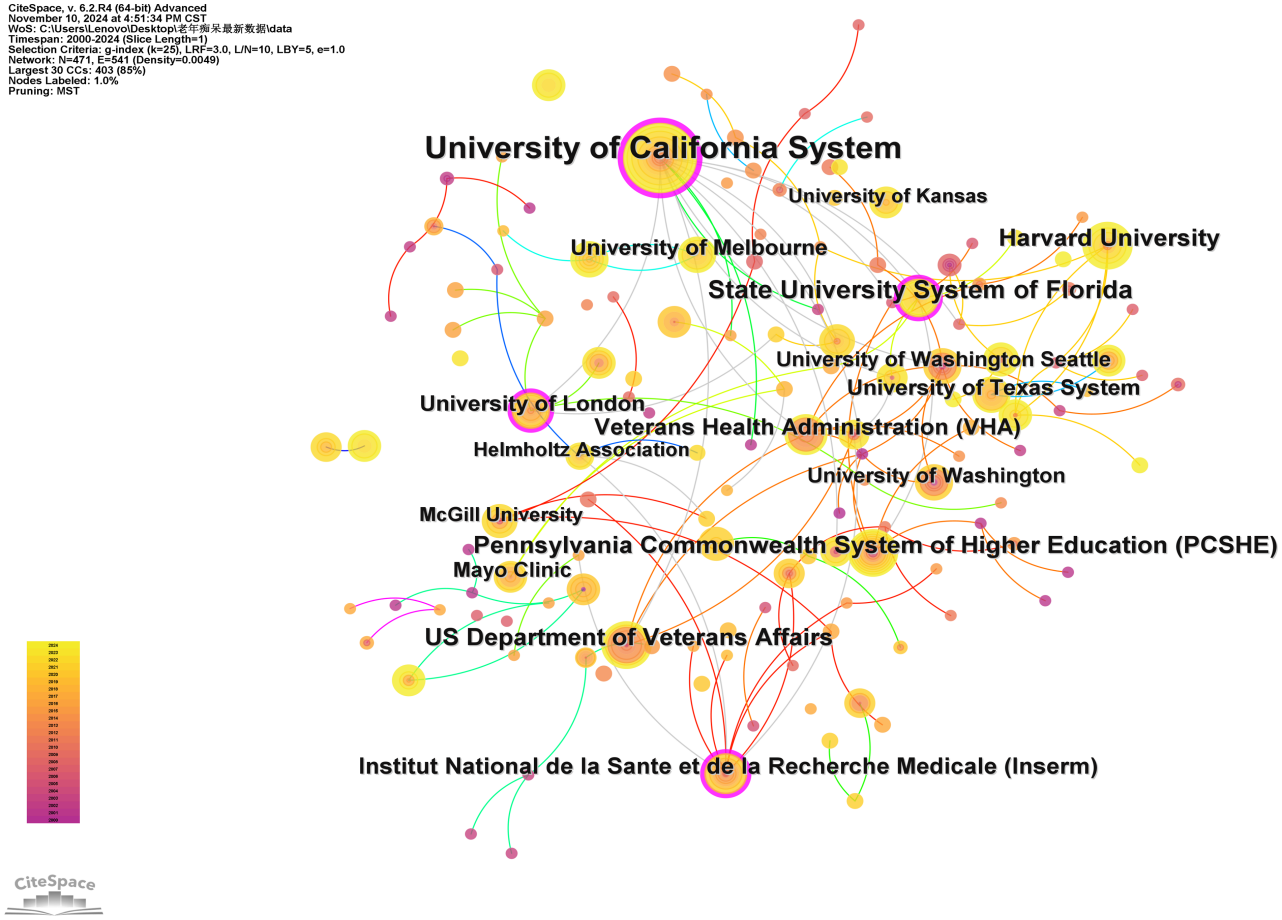
Visualization of AD exercise therapy research institutions.

The field of exercise therapy research for AD exhibits a notable clustering of institutions (Table 3). The University of California system leads with a total of 46 publications (with a centrality of 0.2), but its research contributions are highly concentrated in its medical-focused campuses: the University of California, San Francisco (UCSF) independently published 18 articles, while the Irvine (UCI) and Los Angeles (UCLA) campuses each contributed 9, San Diego (UCSD) contributed 7, and Davis (UCD) and Berkeley (UCB) accounted for only 2 and 1, respectively. This distribution indicates that campuses like UCSF and UCLA are the core drivers of the system’s overall influence, with the output of a single campus (e.g., UCSF’s 18 articles) approaching or exceeding the total output of other systems (e.g., the Florida State University system’s 26 articles). The U.S. Department of Veterans Affairs (30 articles, centrality 0.03) and its subordinate Veterans Health Administration (VHA) (27 articles, centrality 0.14) rank second and third, highlighting the policy priority of AD intervention research for the veteran population. Notably, elite single institutions like Harvard University (27 articles, centrality 0.04) have publication volumes directly comparable to the leading units within the UC system (e.g., UCLA or UCI), while the French National Institute of Health and Medical Research (Inserm) (22 articles, centrality 0.1), though slightly lower in output, demonstrates a higher centrality, indicating its pivotal role in transnational collaborative networks. Among regional university systems, the Pennsylvania State System of Higher Education (26 articles, centrality 0.14) and the Florida State University system (26 articles, centrality 0.02) tie for fourth place, but the former holds stronger academic authority. The University of London (23 articles, centrality 0.09) and the University of Washington (20 articles, centrality 0) further confirm the multipolar landscape of AD research. Additionally, the institution co-occurrence knowledge map (Figure 3) illustrates that both domestic and international institutions have established extensive cooperative relationships, highlighting the global recognition and attention this research has received.

**Table 3:** Top 10 institution on the research of research on exercise therapy for AD.

Rank	Counts	Centrality	Institution	Affiliated principal investigators
1	46	0.2	University of California System	Kristine Yaffe
2	30	0.03	US Department of Veterans Affairs	Craft, Suzanne
3	27	0.14	Veterans Health Administration (VHA)	Borson, Soo
4	27	0.04	Harvard University	Quiroz, Yakeel T.
5	26	0.14	Pennsylvania Commonwealth System of Higher Education	Ganguli, Mary
6	26	0.02	State University System of Florida	Galvin, James E.
7	23	0.09	University of London	Spector, Aimee
8	22	0.1	Institut National de la Sante et de la Recherche Medicale (Inserm)	Vellas, Bruno
9	20	0	University of Washington	Mccurry, Susan M.
10	18	0.05	University of California San Francisco	Chesney, Margaret A.

### 3.5 Analysis of co-citation

Co-citation analysis, which examines the frequency with which two or more documents are cited together by other documents, helps identify significant works and research hotspots within a field (SHGB, 1974). This method uncovers clusters of highly cited documents, offering a succinct overview of thematic research areas related to exercise therapy for AD and enhancing comprehension of key issues.

**Figure 4:**
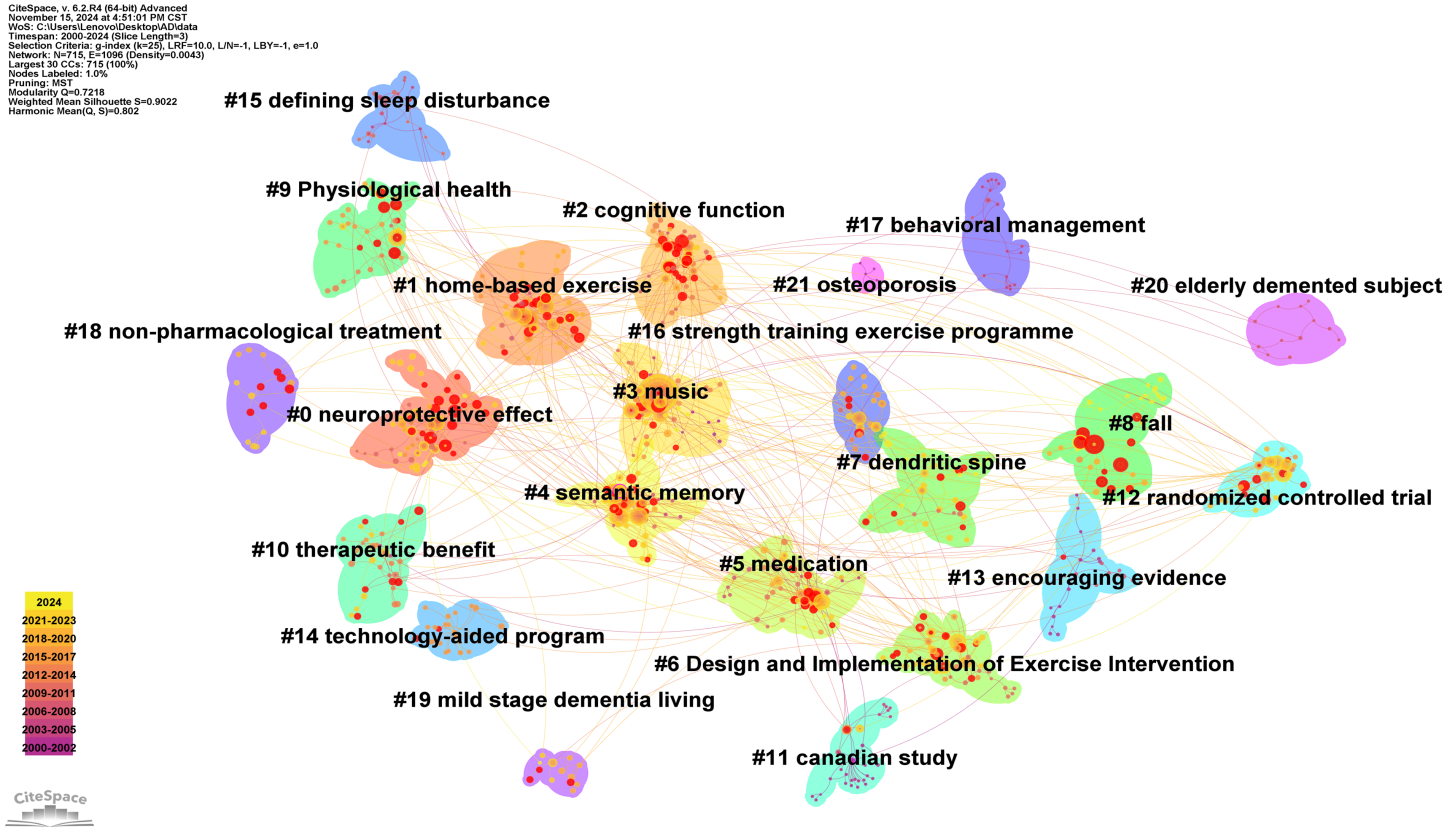
Literature co citation clustering map.

The Q-value of Figure 4 is 0.7218, and its S-value is 0.9022. When conducting bibliometric analysis using CiteSpace, the Q-value and S-value can assist researchers in assessing the rationality of the structure and clustering of the knowledge map, thereby enhancing their understanding of the knowledge structure and development trends within the research field. The Q-value ranges from 0 to 1, with higher values indicating that nodes in the network tend to form tighter communities or modules. An S-value close to 1 suggests excellent clustering results, where data points within their respective clusters are very tightly grouped and show significant differences from other clusters. Therefore, Map 4 demonstrates a significantly reliable clustering structure. Figure 4 contains 21 clusters: #0 neuroprotective effect, #1 home-based exercise, #2 cognitive function, #3 music, #4 semantic memory, #5 medication, #6 Design and Implementation of Exercise Intervention, #7 dendritic spine, #8 fall, #9 Physiological health, #10 Therapeutic benefit, #11 Canadian study, #12 randomized controlled, #13 encouraging evidence, #14 technology-aided program, #15 defining sleep disturbance, #16 strength training exercise programme, #17 behavioral management, #18 non-pharmacological treatment, #19 mild stage dementia living, #20 elderly demented subject, and #21 osteoporosis. By reviewing and summarizing the literature within each cluster, similar clusters were grouped under the same research hotspots, excluding those unrelated to this study. Ultimately, seven main research focuses in the field of exercise therapy for AD since 2000 were identified: the impact of exercise on cognitive function, exercise and music therapy, the combination of exercise and medication therapy, the design and implementation of exercise interventions, exercise and dendritic spines, the effects of exercise on specific symptoms, and exercise and neurophysiological mechanisms. In the discussion section, these seven focus areas will be analyzed in depth.

## 4 Research hotspots analysis

### 4.1 Cognitive function

In Figure 4, #2 “cognitive function” represents a cluster focused on the impact of exercise on cognitive function. As the disease progresses, cognitive decline becomes a critical issue, affecting memory, attention, and executive function. Recent studies increasingly focus on exercise therapy as a potential intervention to improve cognitive outcomes in AD patients (Pahlavani, 2023). Researchers like R-H Harwood have examined the effects of moderate-intensity aerobic exercise on the cognitive function of AD patients. The results indicate that moderate-intensity aerobic exercise can significantly improve memory performance and functional abilities. Notably, optimal effects are observed with sessions of 30 min, less than 150 min per week, up to 3 times weekly (Zhang et al., 2022). While moderate-intensity exercise is generally beneficial, high-intensity exercise may be less effective and potentially detrimental to certain cognitive functions (Liu et al., 2024). Resistance training is particularly effective in slowing overall cognitive decline and improving memory function in AD patients (Lv et al., 2023). Additionally, exercise offers mood-regulating benefits, alleviating symptoms of anxiety and depression, thereby indirectly supporting cognitive function (Pujari, 2024). Exercise also promotes neurogenesis and enhances neuroplasticity by stimulating neurotrophic factors crucial for cognitive function (Wang et al., 2025). Regular moderate-intensity exercise is recommended to maintain and improve cognitive abilities in AD patients, with additional benefits for behavioral and psychological symptoms. Further research is needed to refine exercise protocols and more comprehensively understand their underlying mechanisms.

### 4.2 Exercise and music

In Figure 4, *#2 “music “*is a cluster formed by exercise and music therapy. A large body of research has confirmed that regular physical exercise can significantly improve cognitive function in patients with AD through mechanisms such as promoting hippocampal neurogenesis and increasing the secretion of BDNF (Nascimento et al., 2015). Notably, music therapy, as an independent intervention, has also demonstrated clinical value. Neuroimaging studies have shown that music stimulation can specifically activate the default mode network (DMN) in AD patients, thereby enhancing episodic memory encoding (Sarkamo et al., 2014). Additionally, rhythmic music, by regulating the functional connectivity between the amygdala and the prefrontal cortex, effectively improves emotional regulation disorders (Koelsch, 2014). This independent mechanism of action provides a biological basis for cross-modal interventions. When exercise is combined with music therapy, a significant synergistic effect has been observed. One study tested the effects of music therapy and exercise on the cognitive and motor functions of AD patients, revealing that the combined approach yielded greater improvements in cognitive and motor functions compared to music therapy or exercise alone (Cheour et al., 2023). Experiments conducted by Ghazaleh Shokri et al. further confirmed that the Montreal Cognitive Assessment (MoCA) scores in the combined intervention group increased by 37% more than those in the single-intervention groups, and the improvement in motor function was positively correlated with the aforementioned neural activation. Incorporating cognitive stimuli, such as music, during exercise may induce neural activation and arousal, thereby enhancing both physical and mental performance and maximizing the benefits of physical exercise. Moreover, studies have found that adding a cognitive task during exercise, known as dual-task training, has a more pronounced impact on the physical and mental health of AD patients (Parvin et al., 2020). Further research has revealed the potential of dual-task training in addressing emotional disorders: synchronized musical rhythms during exercise can reduce the amygdala’s sensitivity to anxiety-provoking stimuli (Seinfeld et al., 2013). These findings suggest that the synergistic effects of music-exercise dual-channel interventions offer an innovative therapeutic approach for emotional disorders such as anxiety.

### 4.3 Combination of exercise and medication therapy

In Figure 4, *#5 medication, #10 Therapeutic benefit*, and *#18 non-pharmacological treatment* highlight the emerging research focus on combining exercise with pharmacological interventions specifically for AD. This integrated approach directly targets multiple AD pathological mechanisms simultaneously, providing more comprehensive therapeutic benefits than either intervention alone. Recent AD-specific research demonstrates the synergistic effects of combined exercise and medication therapy across several key pathological domains. Cho et al. (2010) investigated the combination of exercise with *α*-lipoic acid, a potent antioxidant, in AD models and found this combined approach significantly improved spatial learning and memory compared to either intervention alone. The mechanism underlying this synergistic effect was specifically linked to increased levels of BDNF and glucose transporter proteins—both critically impaired in AD pathology. This combination therapy directly addressed the oxidative stress component of AD while simultaneously enhancing neuronal metabolism and plasticity in regions most affected by the disease. Further advancing our understanding of combination therapies in AD, Tao et al. (2023) examined the effects of combining luteolin (a flavonoid with anti-inflammatory properties) with regular exercise in AD model mice. Their findings revealed that this combination significantly improved cognitive performance through complementary mechanisms: reducing neuroinflammation and enhancing autophagy specifically in the hippocampus and cortex—regions highly vulnerable to AD pathology. These results demonstrate how exercise can potentiate the pharmacological effects of certain compounds by targeting multiple AD pathological processes simultaneously, including the impaired clearance of toxic protein aggregates that characterize the disease.

The clinical relevance of these findings is supported by a 2016 study that compared galantamine monotherapy versus galantamine combined with outpatient cognitive rehabilitation (including physical therapy) in AD patients (Tokuchi et al., 2016). After six months, patients receiving the combination therapy showed significantly greater improvements in both cognitive function (measured by Mini-Mental State Examination and Frontal Assessment Battery) and emotional function (measured by the Apathy Scale) compared to those receiving medication alone. These clinical outcomes provide direct evidence that exercise components enhance the therapeutic efficacy of standard AD pharmacotherapy in real-world clinical settings, addressing both the cognitive and neuropsychiatric symptoms that impact quality of life in AD patients.

Collectively, these AD-focused studies demonstrate that combining exercise with medication therapy creates synergistic effects that address multiple aspects of AD pathophysiology—including oxidative stress, neuroinflammation, autophagy dysfunction, and neurotrophic factor deficiency. This integrated approach represents a promising direction for comprehensive AD management that targets the complex, multifaceted nature of the disease through complementary neurobiological pathways, potentially offering more effective therapeutic outcomes than conventional single-modality treatments.

### 4.4 Design and implementation of exercise intervention

In Figure 4, *#1 home-based exercise, #6 Design and Implementation of Exercise Intervention, #14 technology-aided program, and #16 strength training exercise programme* represent clusters related to the design and implementation of exercise interventions. Recent studies have emphasized the crucial role of structured exercise programs in providing comprehensive benefits for AD patients. A meta-analysis published in 2023 examined various exercise modalities and found that resistance training is particularly effective in slowing cognitive decline and improving memory function (Lopez-Ortiz et al., 2021), while multicomponent exercises enhance executive function. This highlights the necessity of tailoring different exercise interventions to specific cognitive outcomes, suggesting that resistance training should be prioritized alongside other modalities to optimize therapeutic effects (Lv et al., 2023). Similarly, a randomized controlled trial focusing on moderate-intensity aerobic exercise demonstrated significant improvements in cognitive assessments and quality of life scores for patients with mild AD who participated in cycling training. Participants in the aerobic group showed increased Mini-Mental State Examination (MMSE) scores and reduced neuropsychiatric symptoms, reinforcing the efficacy of aerobic interventions for cognitive and emotional health (Hoffmann et al., 2016). Another randomized controlled trial investigating high-intensity aerobic exercise showed no significant differences in primary cognitive outcomes between the intervention and control groups. However, it did reveal a notable reduction in neuropsychiatric symptoms favoring the exercise group (Zhang et al., 2022).

The findings from these studies on AD suggest that structured exercise interventions should be personalized based on the patient’s specific cognitive and behavioral symptoms. Resistance training should serve as a core component for improving memory function in AD, while aerobic exercise can be an effective strategy for managing neuropsychiatric symptoms. This targeted approach provides a clear clinical pathway for the comprehensive management of AD, positioning exercise interventions as an evidence-based strategy to enhance cognitive function, alleviate behavioral symptoms, and improve overall quality of life in AD patients.

### 4.5 Exercise and dendritic spines

In Figure 4, *#7 dendritic spine* represents the cluster formed by exercise and dendritic spine formation. The early pathological features of AD are characterized by damage to dendritic structures and a significant reduction in dendritic spine density. Dendritic spines are small, actin-rich protrusions on the dendrites of neurons that form the postsynaptic component of most excitatory synapses in the brain. They play a crucial role in synaptic transmission and plasticity, which are fundamental for higher cognitive functions such as learning and memory (Baylog, 2009; Zagrebelsky et al., 2020). In the course of AD, synaptic degeneration and the cascading decline of neural networks progressively worsen, with the loss of dendritic spine stability being a core driving factor in this pathological process (Peng et al., 2022). Therefore, targeting the delay of synaptic loss and increasing dendritic spine density has become a key strategy for reversing AD cognitive impairment. Exercise intervention can reverse this pathological process. Research has demonstrated that exercise can increase dendritic spine density and prevent memory decline (Mu et al., 2022). A study tested the effects of different exercise intensities on a treadmill on synaptic function in mice. The results showed that both low and moderate intensity exercise increased dendritic spine density, with moderate intensity having the most significant effect (Guo et al., 2023). Another study on mice found that aerobic exercise increased dendritic spine density and prevented synaptic loss. Further findings suggested that exercise might promote synaptic growth and prevent synaptic loss by regulating G-protein-coupled receptors to activate the cyclic AMP/protein kinase A signaling pathway and inhibit synaptic phagocytosis (Yang et al., 2023). Therefore, utilizing the beneficial effects of exercise on dendritic spines, engaging in moderate-intensity aerobic exercise may be helpful in the therapy of AD patients.

### 4.6 The impact of exercise on specific symptoms

In Figure 4, *#8 fall*, *#15 defining sleep disturbance*, and *#21 osteoporosis* represent clusters formed by exercise and specific symptoms. AD is a neurodegenerative disorder, with affected individuals often experiencing sleep disturbances and gait instability, which can reduce quality of life and potentially accelerate disease progression. Pharmacological treatments frequently prove ineffective or cause adverse reactions, sparking interest in non-pharmacological interventions such as exercise. Recent research highlights the impact of exercise on sleep dysfunction in neurodegenerative diseases like Alzheimer’s and Parkinson’s disease. Evidence suggests that exercise can improve sleep disturbances and reduce the progression of neurodegenerative diseases, particularly AD (Memon et al., 2020). Nascimento et al. (2014) evaluated the effects of a multimodal exercise program (including warm-up, muscular resistance, balance and motor coordination, and aerobic fitness) on sleep quality and daily life performance in clinically diagnosed Alzheimer’s patients. The results indicated that regular participation in mild to moderate intensity multimodal physical exercise over six months helped alleviate sleep disturbances and enhance daily living abilities. Additionally, a cross-sectional study involving 114 Alzheimer’s patients found that sleep disturbances were associated with stress and falls, while falls were linked to ongoing medical visits, a history of dementia, and sleep disturbances. Regular exercise was shown to reduce the risk of falls, sleep disturbances, and cognitive impairment, and avoiding stressful situations could mitigate sleep disturbances (Camara-Calmaestra et al., 2023). In conclusion, the beneficial effects of physical activity on overall health are well-documented. In light of the limited efficacy of pharmacological treatments, there is a need to expand the use of non-pharmacological interventions, such as exercise.

### 4.7 Exercise and neurophysiological mechanism

In Figure 4, *#0 neuroprotective effect* represents a cluster formed by exercise and neurophysiological mechanisms. Recent research highlights that physical exercise exerts neuroprotective effects in AD through multifaceted neurophysiological mechanisms, with glycogen synthase kinase-3 (GSK-3) serving as a key molecular mediator. Glycogen synthase kinase- 3 (GSK3) was first identified in the 1980s as a protein kinase responsible for phosphorylating and deactivating glycogen synthase in rabbit skeletal muscle (Embi et al., 1980). GSK3 has been recognized as an evolutionarily conserved Ser/Thr protein kinase with a multitude of substrates. There are two highly similar GSK-3 isoforms in mammals, GSK-3α and GSK-3*β* (Soutar et al., 2010). GSK-3β is the major kinase responsible for the hyperphosphorylation of tau protein, leading to the formation of neurofibrillary tangles (NFTs), a hallmark of AD (Kettunen et al., 2015). As a core regulatory factor in AD pathology, its dysregulated activity drives disease progression by promoting abnormal Tau phosphorylation, exacerbating A*β* production, and impairing neurogenesis and synaptic function (Lauretti et al., 2020). Studies indicate that exercise interventions synergistically inhibit GSK-3β activity through multiple pathways, directly intervening in disease progression. At the Tau pathology level, exercise enhances the phosphatidylinositol 3-kinase (PI3K)/Akt signaling pathway, inducing serine 9 phosphorylation (inhibitory modification) of GSK3-β while reducing tyrosine 216 phosphorylation (activating modification), thereby significantly lowering abnormal Tau phosphorylation and delaying neurofibrillary tangle formation (Han et al., 2024; Ye et al., 2024). This mechanism not only blocks the self-amplifying cycle of Tau pathology but also attenuates the synergistic toxic effects of Tau-mediated amyloid beta (Ittner and Götz, 2011). In the amyloid beta pathology direction, GSK3-β promotes abnormal cleavage of amyloid precursor protein (APP) by activating β-secretase (BACE1), while exercise’s inhibitory effects reduce amyloid beta production and alleviate its hippocampal deposition (Han et al., 2024). Simultaneously, exercise enhances insulin signaling through the PI3K/Akt pathway, repairing Aβ-induced synaptic dysfunction and maintaining hippocampal network integrity (Isla et al., 2016; Kim et al., 2022). Notably, exercise’s regulation of neuroplasticity further amplifies its therapeutic effects: by activating adult neurogenesis in the hippocampal dentate gyrus and upregulating synaptic-related proteins (e.g., PSD-95), exercise can partially reverse synaptic loss and cognitive decline caused by GSK3-β abnormalities (Zang et al., 2017). These mechanisms suggest that exercise does not intervene in a single pathological marker in isolation but rather targets GSK3-β as a molecular hub, simultaneously inhibiting Tau-amyloid beta synergistic toxicity and rebuilding hippocampal neuroplasticity, thereby achieving systemic regulation of the AD pathological network. This multi-target integrated effect provides molecular-level theoretical support for the neuroprotective role of exercise therapy and points the way for the development of non-pharmacological intervention strategies.

## 5 Emerging trends

### 5.1 Analysis of keyword bursts

Keywords are the essence and focal points of an article encapsulating its content and key themes. Keyword burst detection is a widely used method in textual data analysis designed to identify instances where the frequency of one or more keywords suddenly increases within a specified time frame. This method can reveal emerging trends within the data making it crucial for understanding the developmental dynamics of a field and predicting future trends (Webofscience, 2024).

**Figure 5:**
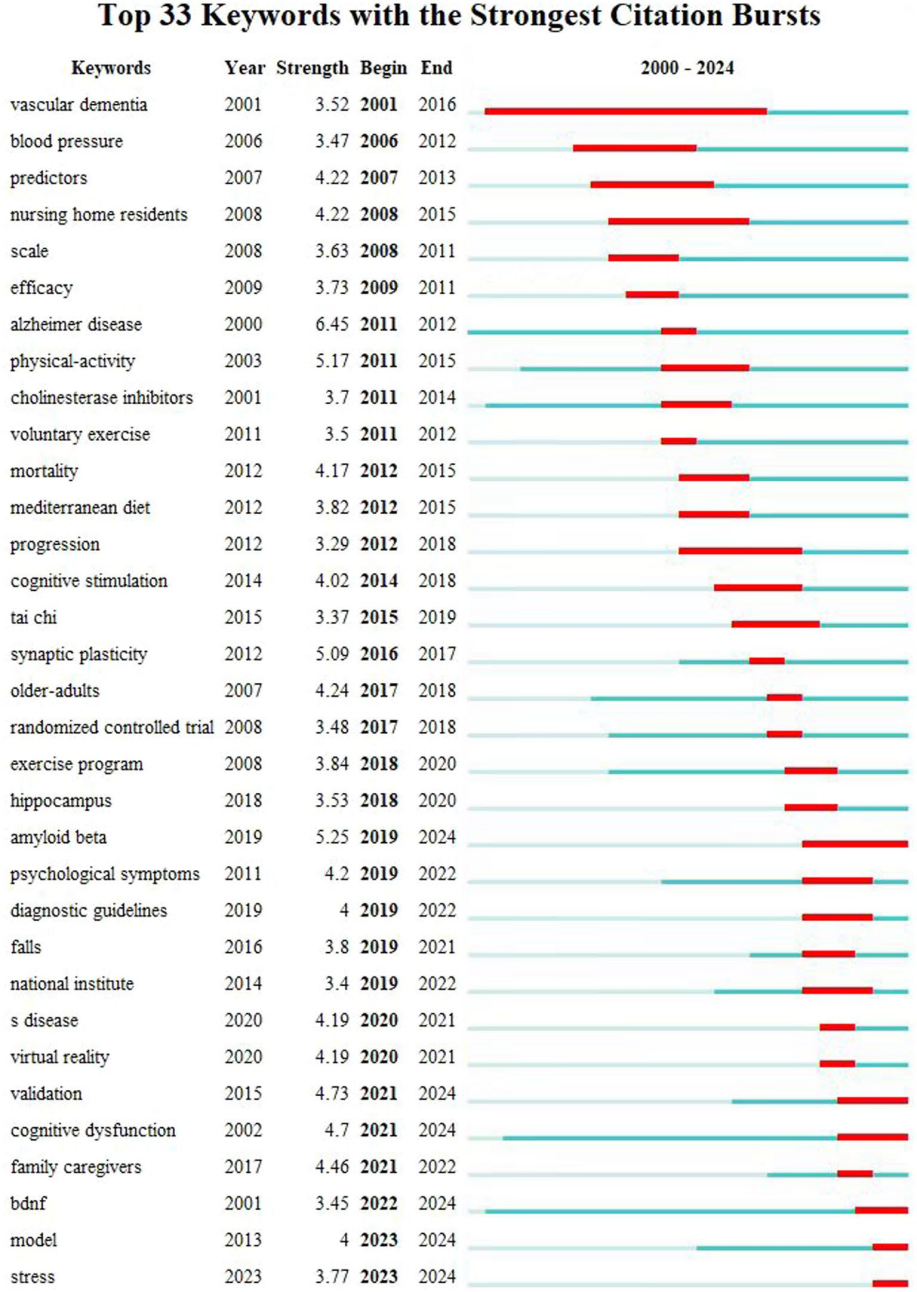
Analysis of keyword bursts in exercise therapy for AD.

As shown in Figure 5, there are six keywords that have experienced explosive citations and continue to be cited until 2024. After summarizing and screening each keyword, it is ultimately determined that emerging trends in the field of exercise therapy for AD may focus on three directions: cognitive dysfunction, BDNF, and Amyloid beta, as well as stress and neuroinflammation. Through in-depth analysis of these three directions, we hope to make new discoveries and provide references for scholars in this field.

### 5.2 Cognitive dysfunction

As shown in Figure 5, research related to cognitive dysfunction has surged since 2021, indicating that this area has become a frontier in AD intervention studies. Given that progressive cognitive decline is a core feature of AD, verifying the effects of exercise therapy based on standardized cognitive assessments has become a research focus in recent years. A meta-analysis demonstrated that aerobic or resistance training interventions significantly improved cognitive abilities in older adults, as measured by the Mini-Mental State Examination (MMSE), Montreal Cognitive Assessment, and other cognitive tests (Xu et al., 2023). In an intervention involving acute aerobic exercise for 79 patients with moderate AD, cognitive monitoring using the Tower of Hanoi, Digit Span, and Stroop tasks revealed that aerobic exercise may help improve cognitive function in AD patients (Ayed et al., 2021). Specifically, aerobic exercise sessions lasting 30 min, less than 150 min per week, up to 3 times weekly, had a significant impact on improving MMSE scores in AD patients (Zhang et al., 2022). A meta-analysis exploring the effects of resistance exercise on cognition showed positive effects on composite cognitive scores (SMD 0.71, 95% CI 0.30–1.12), screening measures of cognitive impairment (SMD 1.28, 95% CI 0.39–2.18), and executive functions (SMD 0.39, 95% CI 0.04–0.74) (Landrigan et al., 2020). Additionally, studies have found that aerobic exercise results in more significant improvements in overall cognition on the ADAS-Cog scale compared to resistance training (Ding et al., 2025). These findings collectively support embedding standardized cognitive assessments within exercise intervention frameworks, enabling precision and quantifiable outcomes in AD treatment strategies.

### 5.3 BDNF and amyloid Beta

As shown in Figure 5, there has been an explosive increase in citations for BDNF and amyloid beta in recent years. BDNF is a member of the structurally and functionally homologous neurotrophic family which also consists of nerve growth factor (NGF), neurotrophin-3 (NT-3) and neurotrophic factor-4/5 (NT-4/5) (Connor et al., 1997). Amyloid beta is an intricate molecule that interacts with several biomolecules and/or produces insoluble assemblies and eventually the nonphysiological depositions of its alternate with normal neuronal conditions leading to AD (Uddin et al., 2020). A study found that both exercise training and chronic BDNF injection can reduce BACE1 activity while increasing ADAM10 activity, thereby reducing amyloid beta production (Uddin et al., 2020). This suggests that increasing BDNF levels through exercise or direct supplementation can reduce amyloid beta production (Baranowski et al., 2023). The mechanism behind this reduction involves decreasing amyloidogenic processing and promoting the non-amyloidogenic cleavage of APP (Endres and Fahrenholz, 2012; Wang et al., 2018). This indicates that enhancing BDNF levels through exercise or direct supplementation can reduce amyloid beta production. The mechanism behind this reduction is achieved by decreasing amyloidogenic cleavage and promoting non-amyloidogenic cleavage of APP. Similarly, another study on AD mice found that exercise induces an upregulation of BDNF levels, which in turn reduces amyloidogenic amyloid beta levels. This emphasizes that exercise not only enhances BDNF expression but also prevents synaptic dysfunction caused by amyloid beta (Jaberi and Fahnestock, 2023). Various mediators induced by BDNF during exercise, including osteocalcin and lactate, highlight that increased BDNF levels can counteract the harmful effects of amyloid beta accumulation on neuronal health. Exercise has a beneficial impact on BDNF levels, supporting cognitive function and neuronal health in AD patients. It also helps reduce amyloid beta levels, although results may vary. These findings highlight the potential of exercise as a non-pharmacological intervention to alleviate some pathological features of AD. Further research is needed to fully understand the mechanisms and optimize exercise protocols for maximum benefit.

### 5.4 Stress and neuroinflammation

Exercise therapy is recognized as an effective intervention for managing stress and cognitive impairment in AD patients. Research indicates that stress induces excessive activation of microglia, leading to reduced microglia–neuron connections and the release of pro-inflammatory cytokines. This negatively impacts synaptic plasticity and neural connectivity, causing neuroinflammation (McDermott et al., 2024; Picard et al., 2021). Persistent neuroinflammation is a hallmark of AD, contributing to memory loss and cognitive decline (Kocamer and Aslan, 2024; Si et al., 2023). Understanding how exercise influences these factors is crucial, given the complex interactions between stress, cognitive decline, and neurodegenerative diseases. A study examined the effects of long-term treadmill training on anxiety and depression behaviors in transgenic AD rats (Wu et al., 2020; Si et al., 2023). Researchers found that exercise significantly alleviated stress-related anxiety and depression, enhancing cognitive abilities and overall well-being. Exercise also counteracts the negative effects of amyloid-beta accumulation and tau hyperphosphorylation by reducing neuroinflammation and oxidative stress, providing neuroprotection. Another study focused on the impact of aerobic exercise on reducing stress and improving cognitive function (Zhang et al., 2022). Results showed that participants engaging in regular aerobic activity had significantly lower cortisol levels (a key biomarker of stress), along with improvements in memory and executive function. This suggests that aerobic exercise could be a powerful tool for alleviating stress-related cognitive decline in AD patients. Further research analyzed how exercise-induced increases in brain-derived neurotrophic factor (BDNF) enhance resilience to stress (Pahlavani, 2023). Findings indicated that higher BDNF levels are associated with improved synaptic plasticity and neuron survival, which are crucial for maintaining cognitive function under stress. Additionally, a study discovered that exercise mimetics—molecules that simulate the beneficial effects of physical exercise, such as BDNF, FNDC5, Gpld1, SAM and microRNAs (miRNA)—can effectively alleviate neuroinflammation and AD (Zhao, 2024). This offers a useful alternative for AD patients unable to engage in regular physical exercise.

In summary, these findings demonstrate that exercise not only enhances cognitive abilities but also helps reduce stress levels, thereby improving the overall quality of life for AD patients.

## 6 Conclusion

The number of articles on exercise therapy for AD shows an increasing trend, with Hauer, Klaus, and Yu, Fang being the most prolific authors in this field. Vellas, Bruno, and McCurry, Susan M are the most frequently cited by Hauer, Klaus, and Yu, Fang. The United States has the highest publication volume, with the University of California leading among institutions. Co-citation clustering reveals that since 2000, research on exercise interventions for AD has focused on seven main areas: the impact of exercise on cognitive function, exercise combined with music therapy, exercise and pharmacotherapy, design and implementation of exercise interventions, exercise and dendritic spines, the impact of exercise on specific symptoms, and exercise and neurophysiological mechanisms. Additionally, an analysis of keyword bursts identified three emerging trends: the impact of exercise on cognitive dysfunction in AD patients, the effects of exercise on BDNF and amyloid beta, and the mechanisms by which exercise alleviates stress in AD patients.

In summary, this study provides a comprehensive review of the field of exercise therapy for AD since 2000, analyzing major contributors (authors, countries, institutions) and identifying current research hotspots and emerging trends. These findings aim to guide future research in the field and offer valuable insights for researchers and clinicians.

## Data Availability

The original contributions presented in the study are included in the article/supplementary material, further inquiries can be directed to the corresponding author/s.
